# Improving Fmoc Solid Phase Synthesis of Human Beta Defensin 3

**DOI:** 10.3390/ijms232012562

**Published:** 2022-10-19

**Authors:** Aleksandra Walewska, Paulina Kosikowska-Adamus, Marta Tomczykowska, Bartosz Jaroszewski, Adam Prahl, Grzegorz Bulaj

**Affiliations:** 1 Department of Organic Chemistry, Faculty of Chemistry, University of Gdansk, 80-308 Gdansk, Poland; 2 Department of Medicinal Chemistry, College of Pharmacy, University of Utah, Salt Lake City, UT 84112, USA

**Keywords:** fmoc solid phase synthesis, cys residue protecting groups, pseudoproline dipeptides, disulfide bond, human beta defensin 3, selenocysteine

## Abstract

Human β-defensin 3, HBD-3, is a 45-residue antimicrobial and immunomodulatory peptide that plays multiple roles in the host defense system. In addition to interacting with cell membranes, HBD-3 is also a ligand for melanocortin receptors, cytokine receptors and voltage-gated potassium channels. Structural and functional studies of HBD-3 have been hampered by inefficient synthetic and recombinant expression methods. Herein, we report an optimized Fmoc solid-phase synthesis of this peptide using an orthogonal disulfide bonds formation strategy. Our results suggest that utilization of an optimized resin, coupling reagents and pseudoproline dipeptide building blocks decrease chain aggregation and largely improve the amount of the target peptide in the final crude material, making the synthesis more efficient. We also present an alternative synthesis of HBD-3 in which a replacement of a native disulfide bridge with a diselenide bond improved the oxidative folding. Our work enables further biological and pharmacological characterization of HBD-3, hence advancing our understanding of its therapeutic potential.

## 1. Introduction

Antimicrobial peptides are important components of the host defense system against diverse pathogens. β-Defensins are disulfide-rich peptides which possess both antimicrobial and immunomodulatory activities. Structural, functional and pharmacological characterization of β-defensins is often hampered by synthetic and recombinant expression methods. In this study, we selected HBD-3 as a model β-defensin since this peptide exhibits strong antimicrobial activity against bacteria that resist to conventional antibiotics: *Staphylococcus aureus*, *Enterococcus faecium* and *Acinetobacter baumannii* [1,2] as well as displays the antifungal activity against *Candida albicans* [3]. Compared to β-defensins HBD-1 and HBD-2, HBD-3 exhibits a broader spectrum of antimicrobial activity [4], properties. HBD-3 was also shown to be an endogenous adjuvant [5], producing immunoprotective effects against HIV [6], preventing binding and entering of HSV [7] and blocking of viral fusion of IAV [8].Therefore, HBD-3 is an attractive molecule for the design and testing of novel analogues with antimicrobial, antifungal and antiviral properties. Additionally, HBD-3 is a useful model to study the structure–function interactions between defensins and various molecular targets, such as chemokine receptor type 4 (CXCR4), potassium channels, or melanocortin receptors (MCRs). HBD-3 can inhibit CXCR4 signaling and in consequence block HIV replication by direct binding of virions [9]. Moreover, recent studies have shown that HBD-3 blocks Kv1.2 and Kv1.3 potassium channels with moderate blocking affinities [10]. This peptide is also a neutral antagonist of MC1R and MC4R blocking the activity of the agonist, such as α-melanocyte stimulating hormone or the inverse agonists such as Agouti signaling protein (Asip) and Agouti-related protein (Agrp) [11].

**Figure 1:**
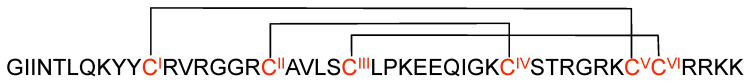
Sequence and disulfide bond connectivity of native HBD-3. Respective Cys residues (red color) are identified with roman numbers.

Cysteine residues play critical roles in the oxidative folding, structural stability of polypeptides, catalysis, response to oxidative stress, metal binding, and cell signaling [12,13,14,15,16,17]. Efficient chemical synthesis of disulfide-rich peptides is critical for drug discovery and lead optimization studies, yet in many cases is a bottleneck because of low yields of forming the correct disulfide bridges. Numerous research groups have taken steps to develop efficient chemical synthesis and in vitro oxidative folding [18,19,20,21,22,23,24,25,26,27]. HBD-3 also belongs to a group of disulfide-rich peptides, stabilized by three disulfide bridges, with pairing as follows: Cys^I^–Cys^IV^, Cys^II^–Cys^V^, Cys^III^–Cys^VI^ (Figure 1) [28]. This peptide can also form dimers in solution, but higher-order oligomers of its structure have not been reported [29,30].The yield of in vitro oxidative folding of HBD-3 (under various oxidation conditions) is low, as a consequence of forming scrambled isomers instead of a correctly folded native peptide [31]. On the other hand, orthogonal oxidative folding can control the formation of intramolecular disulfide bonds which ensures the correct disulfide connectivity, but the processes need to be optimized to obtain reasonably good yield of the native peptide.

## 2. Results

### 2.1. Chemical Synthesis of a Linear Precursor of HBD-3

The main purpose of this studies was to find a feasible and most efficient strategy for solid phase synthesis and oxidative folding of disulfide-rich peptide, human β-defensin 3. 

At first, we synthesized a linear precursor of HBD-3 with all six Cys protected with a trityl group. The oxidative folding of this peptide with the reduced and oxidized glutathione resulted in a mixture of different products. The native HBD-3 could not be properly identified, because two of the products had the same mass, expected for the native HBD-3 (calc. 5155.2 Da). Due to challenges with the direct oxidative folding, we decided to synthesize HBD-3 with orthogonal groups of Cys residue to facilitate the native-like disulfide bonds connectivity.

In a second attempt to optimize chemical synthesis we synthesized two linear precursors of HBD-3 with different Cys protection group pattern (Figure S1). This synthesis was carried on Fmoc-Lys(Boc)-Wang resin. These two peptides had β-thiol groups of Cys^18^ and Cys^33^ protected with trityl group. In the amino acid sequence of HBD-3 both Cys^18^ and Cys^33^ residues are comparatively close to each other; therefore, formation of this disulfide bond as first selection was clear. The second pair of Cys residues with Acm groups were on Cys^11^, Cys^40^ (namely peptide 1) and on Cys^23^, Cys^41^ (namely peptide 2). The third pair of Cys residues with Mob group was applied to Cys^11^, Cys^40^ (peptide 2) and Cys^23^, Cys^41^ (peptide 1) (Figure S1). It appeared that selective oxidative folding of peptide 2 was unproductive. After forming of the second disulfide bond, between Cys^23^ and Cys^41^, we obtained less than 1 mg of this peptide. This small amount of peptide 2 prevented from processing of removal of Mob group and closing the last disulfide bond. Nevertheless, the arrangement of Cys residue protection groups of peptide 1 was successful and following peptide synthesis was designed to have the same order of Cys residue protection groups.

**Figure 2:**
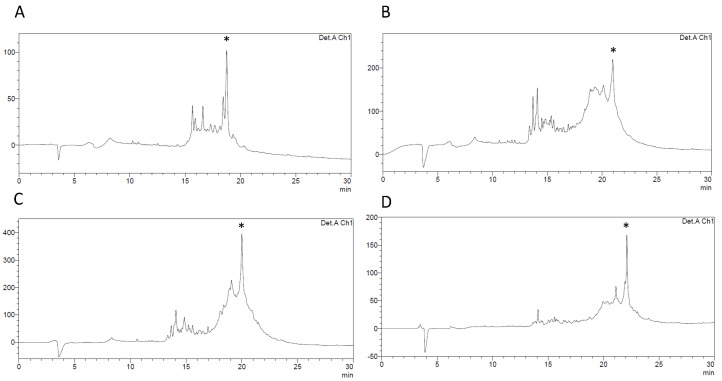
RP-HPLC traces of crude, linear precursor of HBD-3 synthesized on various resins: (**A**) HBD-3 synthesized on Fmoc-Lys(Boc)-TentaGel S PHB resin; (**B**) HBD-3 synthesized on Fmoc-Lys(Boc)-Wang resin; (**C**) HBD-3 synthesized on Fmoc-Lys(Boc)-HMPB-ChemMatrix; (**D**) HBD-3 synthesized on Fmoc-Lys(Boc)-HMPB-ChemMatrix and with three pseudoproline building blocks; The asterisk indicates the linear HBD-3 in the crude material.

In the next approach, we synthesized linear precursors of HBD-3 using three different resins: Fmoc-Lys(Boc)-Wang with loading of 0.57 mmol/g, Fmoc-Lys(Boc)-TentaGel S PHB with substitution of 0.19 mmol/g and Fmoc-Lys(Boc)-HMPB-ChemMatrix with loading of 0.40 mmol/g. Chemical synthesis with different resins showed that using Fmoc-Lys(Boc)-Wang resin gave poor results (Figure 2B). The application of Fmoc-Lys(Boc)-TentaGel S PHB resin as well as Fmoc-Lys(Boc)-HMPB-ChemMatrix resin was more successful (Figure 2A,C, respectively). Low-loaded resins are suggested for synthesis of long and difficult sequences. The lowest-loading TentaGel S PHB resin yielded 22.2% of pure linear HBD-3. However, in our case, the type of core matrice used in HMPB-ChemMatrix resin was the most efficient. Thus, subsequent syntheses were prepared on the HMPB-ChemMatrix resin, as this resin appeared to provide the best yield of all three: 25.5% (Table 1). MALDI-TOF analysis confirmed that experimental masses corresponded to expect linear precursor of HBD-3.

The next synthesis conditions tested, coupling reagents, confirmed that optimizing reagents for synthesis of this long and easy to aggregate peptide, is important. Chemical synthesis of 45-amino acid sequence of HBD-3 is problematic, so to increase the yield of a final product we applied PyOxim as an alternative coupling reagent to benzotriazoles. Unfortunately, this approach was unsuccessful. In the crude mixture there was no expected product, only combination of other by-products. Nevertheless, using of HATU and HOAt as coupling reagents was effective and since they are often successfully used reagents, further changes were not made in this aspect of the chemical synthesis. In the next step, we decided to apply additional modifications in the chemical synthesis of HBD-3. Using Aggrescan server we were able to check the “weak spots” (Figure S2) in the amino acid sequence of HBD-3 which may aggregate and prevent from the proficient coupling of subsequent amino acid residues [32]. We modified the solid-phase synthesis introducing pseudoproline dipeptides into “weak spots” that were detected by Aggrescan. The next linear precursor of HBD-3 was synthesized with one pseudoproline block instead of Ser^34^ and Thr^35^ residues incorporated in place of β-turn. This single modification turned out to be ineffective. During the following synthesis we applied three pseudoproline dipeptides into sequence of HBD-3 (Figure 2D). This method emerged to be more efficient. After RP-HPLC purification of the crude product we obtained 38.4% yield of a linear precursor of HBD-3. As a result, the chemical synthesis of HBD-3 was set up on Fmoc-Lys(Boc)-HMPB-ChemMatrix and with three pseudoproline blocks as the best possible option.

### 2.2. Oxidative Folding with the Orthogonal Thiol Trt/Acm/Mob Strategy

**Figure 3:**
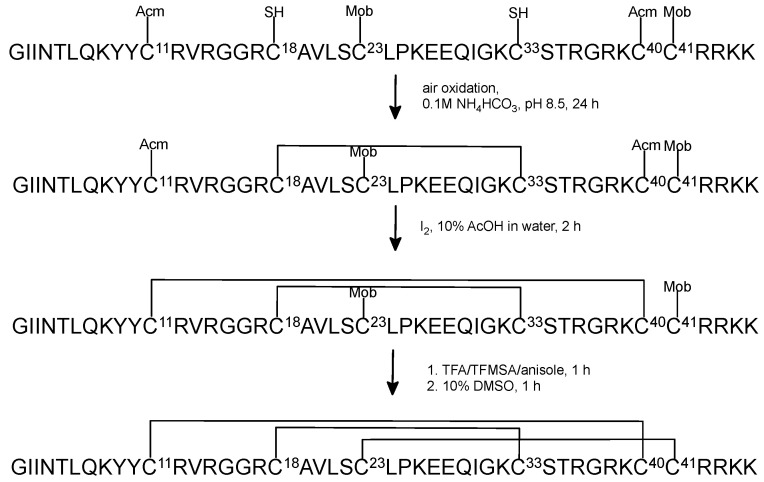
The scheme of regioselective oxidative folding of HBD-3 with Trt/Acm/Mob orthogonal group pattern.

When optimization process of chemical synthesis was completed, we employed the orthogonal Trt/Acm/Mob strategy for Cys residue protection for synthesis of HBD-3. The peptides were cleaved from the resin with simultaneous removing of protecting groups, except for Cys(Acm) and Cys(Mob) (Figure 3). The first oxidative folding step was prepared in the presence of 0.1 M NH_4_HCO_3_ buffer. The monitoring of the progress of the reaction by RP-HPLC and mass spectrometry showed that after 24 h the first disulfide bond was effectively formed. At this point the peptide was not purified before the next step, because our previous laboratory experiments indicated that both purified and not purified peptide with the first disulfide bond closed produced the same yield of removing Acm group and formed the second disulfide bond (Figure S3).

The second oxidative folding step was carried out with iodine solution to remove acetamidomethyl (Acm) group and to form the second disulfide bond. This step required purification of the peptide after the second oxidative folding reaction. This part of the oxidative folding was more problematic due to appearance of by-products of this reaction. The reaction time and the rate of adding iodine solution needed to be optimized. 

The most challenging was removing of Mob group from the third pair of Cys residues and closing the remaining disulfide bond. This reaction required the extraction step after mixing with solution of TFMSA/TFA/anisole in ice bath. It is probable that during the extraction, part of the peptide dissolves in diethyl ether; hence, the final oxidative folding yield is ineffective. The second crucial part of this step was purification of native HBD-3 after closing the last disulfide bridge. The solution after this reaction was diluted. Unfortunately, the low concentration of the peptide in solution affected the purification process on semipreparative column on which the peptide somehow was disappearing. Consequently, we used analytical RP-HPLC system to purify the final peptide. Furthermore, photosensitive DMSO in the solution triggered off that the oxidated product had to be purified over the same day. As a result, the entire process of removing Mob group and closing of the third disulfide bond was problematic, time consuming and lead to poor (3.6%) final yield of the native-like HBD-3 (Table 1). Moreover, in the last purification step it was impossible to obtain a three-disulfide bridged HBD-3 with the highest purity. The MALDI-TOF analysis confirmed the right mass of the native peptide (Figure S4). 

### 2.3. Oxidative Folding with the Orthogonal Thiol Mmt/Trt/Acm Strategy

**Figure 4:**
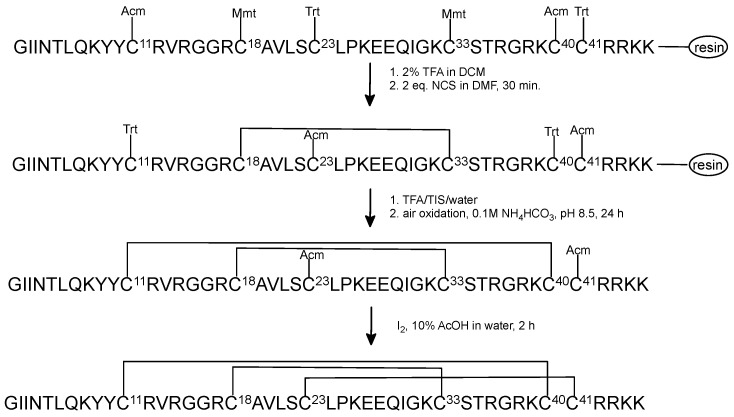
The scheme of regioselective oxidative folding of HBD-3 with Mmt/Trt/Acm orthogonal Cys protection.

Next, we synthesized the linear precursor of HBD-3 with the following orthogonal group pattern: Mmt/Trt/Acm of Cys residues protection (Figure 4). 

**Figure 5:**
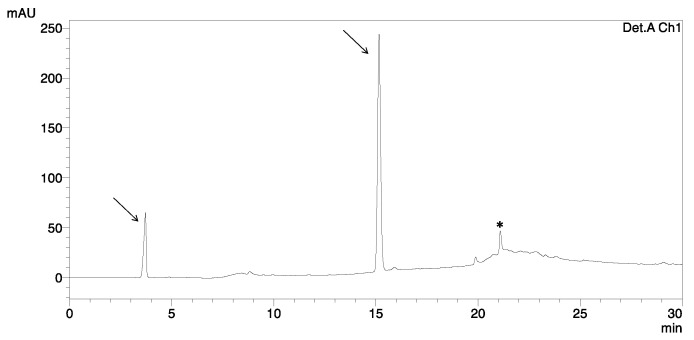
Chromatogram of on-resin NCS oxidation of HBD-3 (marked with asterisk); nonpeptide impurities are indicated by an arrow.

The first disulfide bridge was closed on-resin in two steps, using 2% TFA in DCM to remove Mmt group and NCS to form the disulfide between Cys^18^ and Cys^33^. Removing of Mmt group was carried out by washing the resin with 2% TFA in DCM as mentioned above. The process of Cys deprotection was monitored by changing the color of the solution: yellow–orange–yellow–no color, respectively. The formation of a disulfide bond was completed by adding 2 eq. of NCS in DMF. After removing the peptide from the resin in standard procedure, HPLC analysis showed that peptide aggregated in this reaction and the main product with formed Cys^18^–Cys^33^ bond occurred in a poor yield (Figure 5). Overall oxidative folding of three disulfide bond resulted in a very low yield (less than 0.5%). 

### 2.4. Chemical Synthesis and Oxidative Folding of [Sec^18,33^]HBD-3

**Figure 6:**
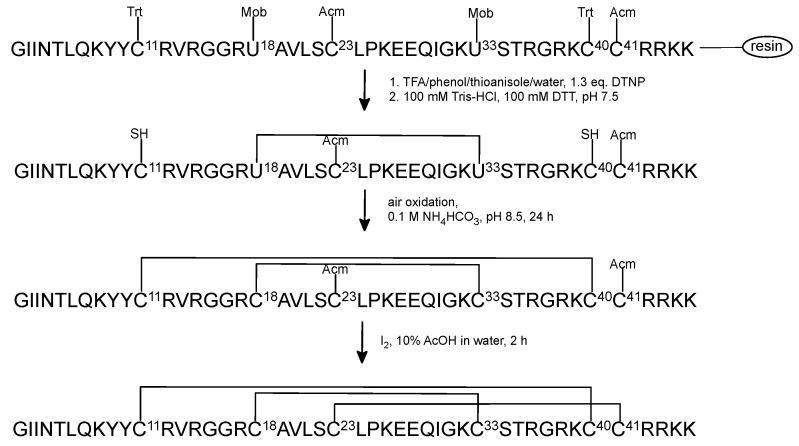
The scheme of oxidative folding conditions of [Sec^18,33^]HBD-3.

The final approach to the optimization of the synthesis and oxidative folding of HBD-3 was isosteric replacement of one native disulfide bond with one diselenide bond. The previous studies indicated that this tactic can be successfully used for disulfide rich peptides. It is noteworthy that disulfide-to-diselenide substitutions did not significantly affect biological activities or native-like conformations of other peptides [33,34,35,36,37,38,39]. In our case Sec^18^ and Sec^33^ were incorporated to the sequence of HBD-3 (Figure 6).

Chemical synthesis, thiolysis reaction, purification of the precursor with the diselenide bond and oxidative folding of the remaining two disulfide bonds appeared to be more efficient compared to the synthesis of native HBD-3 with orthogonal Cys groups. Final product of [Sec^18,33^]HBD-3, confirmed by MALDI-TOF analysis (Figure S3), was obtained with the best, 13.5% yield of the oxidative folding, whereas native HBD-3 with 3.6% yield of the orthogonal formation of all disulfide bridges.

### 2.5. Regioselective Oxidative Folding versus Direct Oxidative Folding of HBD-3

**Figure 7:**
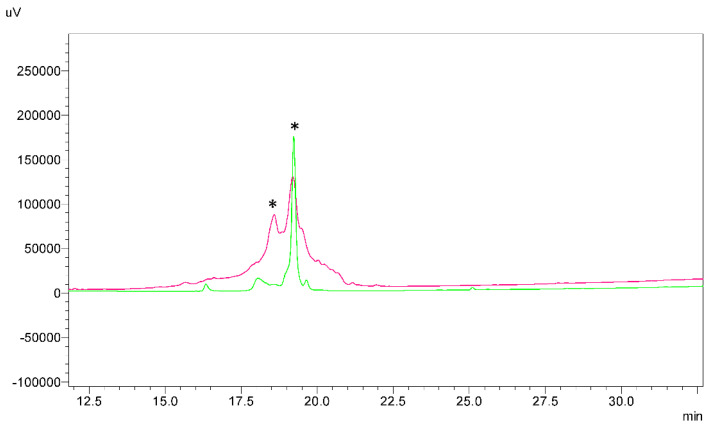
Chromatograms of the folded HBD-3. A green trace denotes the native-like form of HBD-3 #1 synthesized using the orthogonal Cys protection strategy. A pink trace indicates products of the directly folded HBD-3 carried in the presence of 1 mM GSSH and 1 mM GSH, 0.1 M Tris-HCl (pH 7.5). The two main peaks of the directly folded HBD-3, with the same mass as expected for the native HBD-3, are marked with asterisks. The second peak (HBD-3 #2), with related time to the native-like form of HBD-3 #1 was collected and its antimicrobial activity was tested as well as HBD-3 #1.

When we obtained the native HBD-3 which was synthesized with orthogonal Cys groups, in the next step we compared HPLC chromatograms and mass spectrometry data of direct oxidative folding of linear HBD-3 with free six thiols and the native HBD-3 after DMSO oxidation of the third disulfide (Figure 7). We found that the direct oxidative folding with GSSG/GSH resulted in a mixture of different compounds, but we were capable to separate the three main products from the mixture and analyzed them with MALDI-TOF. The two of them (marked with the asterisk in the figure) had the same mass as the native peptide but just one, namely HBD-3 #2 had related time on HPLC to the native HBD-3 (namely HBD-3 #1). The peak of HBD-3 #2 was collected using RP-HPLC and then its antimicrobial activity was tested as well as HBD-3 #1 and [Sec^18,33^]HBD-3.

The final analytical RP-HPLC data and MALDI-TOF analysis of synthesized peptides are shown in Figures S4 and S5 and their purity in Table S1. We assume that the impurities of the final products might derive from the Mob group adducts or iodine adducts that could be present as contaminants after closing of the third disulfide bond. In addition, disulfide-step formation of used synthetic strategies illustrates Figure S6. 

### 2.6. Antimicrobial Activity against E.coli and S. aureus

First, to evaluate the stability of synthesized peptides for anticipated antimicrobial studies, peptides were dissolved in 10 mM PIPES buffer, and their aliquots were collected in different time points. RP-HPLC analysis of collected samples indicated that HBD-3 #1 was stable after 24 h. Chromatograms of HBD-3 #2 and [Sec^18,33^]HBD-3 after 24 h in PIPES buffer showed that native-like peptides were accumulated as main peaks, but minor shoulders peaks were also shown (Figure S7). At this point we decided that PIPES buffer was applicable for the evaluation of bactericidal properties of synthesized peptides. In the next step, the compounds were tested against Gram-negative and Gram-positive bacteria. The antimicrobial activity of peptides is summarized in Table 2. Three tested peptides exhibited activity against Gram-negative bacteria *E. coli* as well as Gram-positive bacteria *S. aureus*. The activity of native HBD-3 #1, HBD-3 #2 and [Sec^18,33^]HBD-3 were comparable. Nevertheless, native HBD-3 #1, which was synthesized with regioselective formation of three disulfide bonds, was more active than HBD-3 #2, which was oxidized directly. HBD-3 #1 and [Sec^18,33^]HBD-3 displayed the same activity against *E. coli*; however, [Sec^18,33^]HBD-3 is less active against *S. aureus.* More details about bactericidal activity of these synthesized peptides will be discuss elsewhere (manuscript in preparation). 

## 3. Discussion

This article describes diverse state-of-the-art strategies of synthesis and the oxidative folding of 45-residue, disulfide rich human β-defensin 3. Many scientists face a dilemma of which synthetic strategy is more efficient: (A) a multi-step and more laborious process, with orthogonal Cys protection groups, or (B) one-step process, with direct oxidative folding, but with a challenge of identifying the correct disulfide pattern and necessity to determine disulfide bond connection. Using of orthogonal strategy for oxidative folding of two-disulfide bonds peptide is only one step longer compared to direct oxidative folding. However, usage of either direct or regioselective procedures of oxidative folding for three-disulfide bond peptides are more complex and hence individual for each peptide.

As a result of the above, we decided to apply further modifications in the synthesis of native HBD-3, namely, to introduce pseudoproline dipeptides into specific positions in the amino acid sequence of HBD-3. In our case, incorporating of pseudoproline dipeptides between Asn^4^ and Thr^5^, Leu^21^ and Ser^22^ and Ser^34^ and Thr^35^ improved the final yield of the chemical synthesis. Especially the first two pseudoprolines in the “weak spots” helped to reach better yield. The use of pseudoproline blocks was intended to prevent the aggregation process, which is very common in the synthesis of long polypeptides such as HBD-3, which contains 45 amino acid residues. In addition, introducing residues of pseudoproline into the amino acid sequence interferes with the formation of a secondary structure formed by intracellular and intermolecular interactions during the lengthening of the polypeptide chain. Therefore, such modifications favorably affect the yield of acylation and Fmoc removal in subsequent synthesis steps and sum up the yield of the final synthesis product. 

The selection of orthogonal synthesis Cys protective groups: Cys^I^–Cys^V^ (Acm), Cys^II^–Cys^IV^ (Trt) Cys^II^–Cys^VI^ (Mob) appeared as the only route in which we obtained the native HBD-3. The second strategy, called as strategy for peptide 2 was insufficient (Figure S1). Removing of Mob groups and forming of the third disulfide bond was impossible due to insufficient quantity of peptide 2 with two disulfide bonds closed. These findings are similar to those reported by Kluver and co-workers, that the following orthogonal groups Cys^I^–Cys^V^ (Acm), Cys^II^–Cys^IV^ (Trt) Cys^III^–Cys^VI^ (t-Bu) employed to shorter version of HBD-3 (without 5 first amino acids (GIINT) at the *N*-terminus) were the only option to obtain final three disulfide bridged peptide with sufficient final yield and product quality [28].

Using 4-methoxytrityl (Mmt) group in a strategy Mmt/Trt/Acm for Cys protection, in our case, seemed to be a great alternative to Trt/Acm/Mob approach. On resin removing of Mmt and forming the first disulfide bond with NCS has been reported as efficient and rapid technique for synthesis two-disulfide bond SI conotoxin [40]. Unfortunately using Mmt protective groups in a long peptide such as HBD-3 leaded to insufficient yield of the reaction. Similar to our findings, Kellenberger et al. showed that applying combination of Trt/Acm/Mob Cys protection groups for PMP-D2, 35-residue insect peptide was more satisfied than Mmt/Trt/Acm strategy [41].

Replacing one native disulfide bond with diselenide bond provided improved oxidative folding of the peptide but slightly worse antimicrobial activity towards *E. coli* and *S. aureus* compared to native HBD-3 #1. On these terms HBD-3 with diselenide bond might be a good alternative to regioselective synthesis of native HBD-3.

Taken together, the chemical methods used in our report show promising results, however the difficulty in obtaining high-purity final products has shown some limitations that need to be addressed in the future.

**Figure 8:**
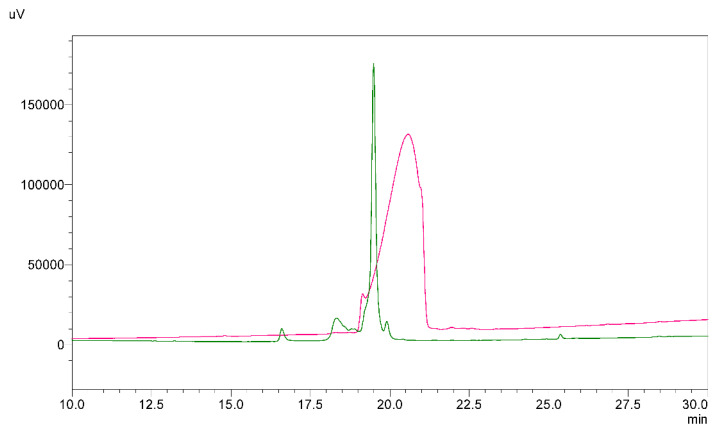
Chromatograms of commercial HBD-3 (pink trace) and HBD-3 #1 synthesized with orthogonal disulfide bond formation strategy (green trace).

As far as we studied published literature, there was no clear study of disulfide bond pattern in recombinant human beta defensins [3]. Occasionally researchers use commercial HBD-3 but usually they do not provide an information about the method of determination of disulfide bonds. In our case commercial native HBD-3 compared to synthesized native HBD-3 #1 with orthogonally closed disulfide bonds had slightly different retention time and much broader peak profile on HPLC (Figure 8). 

Although HBD-3 is a promising drug-lead peptide, there are not many studies related to long, synthetic analogues of this compound [31,42].

The application of antimicrobial peptides (AMPs) which include host defense peptides (CHDP) has been under research investigation for many years. Their wide-ranging functions: antibacterial, antifungal, antiviral as well as immunomodulatory mechanisms are studied with promising results. Numerous CHDP are in different stages of clinical trials, including phase III. Most of them are formulated for topical applications or as inhalants for the infection’s treatment, however there are also selected peptides for oral or intravenous purpose [43,44,45]. 

Many laboratories study a role of HBDs in various cancers. It is shown that HBD-3 is overexpressed in oral squamous cell carcinoma (OSCC) and may play a significant role in oncogenesis [46]. HBD-3 is also overexpressed in cervical cancer [47], but underexpressed in colon cancer [48]. Importantly, many studies shown that various cancers demonstrated unlike expression of HBDs, because of their differential interactions with receptors on cells. These findings indicate that continuation of research on role of HBDs in a particular cancer type may lead to novel ways to use these molecules as potential biomarkers for diagnostic or prognostic purposes [46]. Feng et al. reported that HBD-3 is an endogenous antagonist of CXCR4 by internalization of this receptor without its activation and thus can protect from HIV [9]. In their following studies modified HBD-3 molecules were also successfully used as antagonists of CXCR4 [49]. Therefore, HBD-3 and its analogues may have a novel role in developing new strategy for HIV therapies or immunomodulation. However, to continue these studies it is necessary to synthesize HBD-3 and its derivatives with individual disulfide bond scaffold by using orthogonal protection of Cys residues in order to analyze the structure–function details of HBD-3-CXCR4 antagonism [49]. Our current work suits entirely into this research trends and demonstrates that optimization of chemical synthesis of HBD-3 is required to obtain a good yield and enough amount of this peptide to consider its role in cancer therapies, would healing, angiogenesis, metastasis and fibroblast activation [50]. In addition, our research studies offer advantages of forming specific disulfide bond topology in a chemically synthesized peptide over the recombinant method, in which the resulting disulfide scaffold(s) is (are) difficult to be determined.

## 4. Materials and Methods

### 4.1. The Chemicals

Fmoc-protected amino acids (except of listed below), HATU, HOAt were purchased from GL Biochem (Shanghai) Ltd., Shanghai, China. Fmoc-Cys(Acm)-OH and Fmoc-Cys(Mob)-OH were from Bachem, Bubendorf, Germany. Fmoc-Lys(Boc)-Wang resin, TFA and PyOxim were from Merck, Darmstadt, Germany. Fmoc-Lys(Boc)-HMPA ChemMatrix resin was from PCAS BioMatrix, Inc., St-Jean-sur-Richelieu, QC, Canada. Fmoc-Ser(tBu)-Thr(ψMe,Mepro)-OH, Fmoc-Leu-Ser(ψMe,Mepro)-OH, Fmoc-Asn(Trt)-Thr(ψ^Me,Me^pro)-OH, Cys(Mmt)-OH and Fmoc-Sec(Mob)-OH from Novabiochem, Läufelfingen, Switzerland. Fmoc-Lys(Boc)-PHB resin was purchased from Rapp Polymere, Tübingen, Germany. *N*-chlorosuccinimide was from Sigma-Aldrich, Darmstadt, Germany. Commercial, native HBD-3 was purchased from Peptide Institute, Inc., Osaka, Japan.

### 4.2. In Silico Prediction of HBD-3 Aggregation Potential

The Aggrescan, a server was used to predict and evaluate “hot spots” of aggregations in HBD-3 [32]. The sequence in FASTA format was submitted on 13 September 2017 to: http://bioinf.uab.es/aggrescan/ and then obtained data were analyzed. 

### 4.3. Peptide Synthesis

Peptides were synthesized on an automated peptide synthesizer Symphony, Protein Technologies, Tucson, AZ, USA; using Fmoc/tBu SPPS protocols at 25 μmol scale (optimization process) and 100 μmol scale (final synthesis). Cysteine residues were protected with trityl (Trt) groups, acetaminomethyl (Acm) groups and *p*-methoxybenzyl (Mob) groups or *p*-methoxytrityl (Mmt). The 2.5-fold excess of Fmoc-L-amino acids and either HATU/HOAt or PyOxim were used as well as 5-fold excess of NMM (*N*-methylmorpholine), with DMF as a solvent. Fresh solution of PyOxim/NMM in DMF was preparing every 15 h during the synthesis. Pseudoproline dipeptides as well as Fmoc-Sec(Mob) residue were added manually with a 1.5-fold excess. After chain assembly the peptides were washed with DCM and dried with N_2_. Then, the peptides were cleaved from the resin by a five-hour treatment with a mixture of: trifluoroacetic acid/phenol/1,2-ethanodithiol/triisopropylsilane/water: 90/2.5/2.5/2.5/2.5 by volume. Peptide with Mmt/Trt/Acm Cys protection groups was treated with modified reagent K: TFA/TIS/H_2_O 95/2.5/2.5 by volume. Peptide with the first disulfide bond closed on the resin was treated with modified reagent K: TFA/TIS/H_2_O 95/2.5/2.5 by volume [40]. HBD-3 with selenocysteine residues was cleaved with a mixture of: TFA/phenol/thioanisole/H_2_O 90/2.5/7.5/5 by volume and 1.3 equivalents of DTNP ([2,2′-dithiobis(5-nitropyridine)]) [51]. In the next step, the resin was filtered off, rinsed with additional 1–2 mL of TFA and the crude peptides were precipitated with cold ether diethyl. The resulting solid was centrifuged (4 °C, 7000 RPM, 15 min), washed again with ether, centrifuged, redissolved in water and lyophilized.

To remove the thio-5-nitropyridine derivative from selenocysteine residue and form diselenide bond, the peptide was treated with 100 mM DTT (threo-1,4-dimercapto-2,3-butanediol), 100 mM Tris-HCl, 1 mM EDTA, pH 7.5 at RT. The concentration of the peptide was about 4 mg/mL. After 2 h, formic acid (8% final concentration) was added to quench the reaction. 

### 4.4. Analysis and Purification

The crude peptides were analyzed by analytical RP-HPLC on Jupiter Proteo C12 column (250 × 4.6 mm, particle size 4 μm, Phenomenex, Torrance, CA, USA) using LC20AD Shimadzu system, Kyoto, Japan. Solvent A was 0.1% TFA (*v*/*v*) in water and solvent B was 0.1% TFA (*v*/*v*) in 80% aqueous acetonitrile. Elution was performed with a linear gradient 1–80% of solvent B in 30 min at 1 mL/min flow rate. The HPLC separations were monitored at 226 nm.

Purification of crude peptides, HBD-3 with two disulfide bonds closed or with one diselenide and two disulfide bridges formed were performed on Waters system Milford, MA, USA on semi-preparative Jupiter Proteo C12 column (250 × 10 mm, particle size 4 μm, Phenomenex, Torrance, CA, USA) using linear gradient 10–80% of solvent B over 60 min, at a flow rate 5 mL/min, with UV detection at 226 nm. Final purification step after removing of Mob groups from last two Cys residues and oxidative folding with DMSO was performed on LC-20AT Shimadzu system (Japan) using analytical Jupiter C18 column (250 × 4.6 mm, particle size 5 μm, Phenomenex, Torrance, CA, USA) with linear gradient 1–80% of solvent B in 30 min at 1 mL/min flow rate and UV detection at 226 nm.

Collected fractions were analyzed by analytical RP-HPLC. Fractions of high (<95%) HPLC homogeneity and with expected mass were combined, lyophilized, and used in following experiments.

The molecular weight of the peptides was identified by MALDI-TOF mass spectrometry (Biflex III or Autoflex MaX, Bruker, Bremen Germany).

### 4.5. Oxidative Folding

Formation of the disulfide bond between Cys^18^–Cys^33^ (native HBD-3) or Cys^11^–Cys^40^ ([Sec^18,33^]HBD-3) with free thiol groups was performed in 0.1 M ammonium bicarbonate (NH_4_HCO_3_) buffer, pH 8.5, room temperature, at a peptide concentration of 0.1 mM. The solution was stirred overnight under air oxidation. Progress of the oxidative folding reaction was monitored by analytical RP-HPLC and mass spectrometry. After ~24 h, formic acid (8% final concentration) was added to quench the reaction and the solution was lyophilized. 

Removing of Acm group and forming disulfide bridge between the remaining Cys^11^–Cys^40^ for the native HBD-3 and Cys^23^–Cys^41^ for [Sec^18,33^]HBD-3 were carried out in 10% acetic acid and 5 mM iodine in methanol, at peptide concentration of 0.1 mM. After 2 h the reaction was quenched with 1 M ascorbic acid. The mixture was purified by semi-preparative RP-HPLC, collected fractions were analyzed by analytical RP-HPLC, lyophilized, and followed by mass spectrometry analysis.

To remove the *p*-methoxybenzyl groups from the third pair of cysteine residues, the HBD-3 at a concentration of 3 mg/mL was solved in a cleavage solution of TFMSA/TFA/anisole (1:8:1, by volume) cooled in an ice bath. The reaction was carried out at 0 °C for 1 h in the ice bath. Then, the solution was diluted about 15-fold with iced water and extracted 3 times with diethyl ether. The water layer was recovered and the remaining Cys^23^–Cys^41^ disulfide bond was closed with DMSO (10% of total volume, 1 h) followed by analytical RP-HPLC purification. The identities of the final product, native HBD-3 was confirmed by MALDI-TOF analysis.

On-resin removing of Mmt group was performed with 2% of TFA in DCM. The peptidyl resin was washed several times with deprotection mixture until there was no color of the solution. Subsequently, the peptidyl resin was treated with 2 equivalents of NCS (*N*-chlorosuccinimide) in DMF (150 μL/μmol of peptide). After 30 min, the resin was washed with DMF (5 × 1 min) and DCM (5 × 1 min) and dried out. In the next steps, which are described above, peptide was cleaved from the resin, disulfide between Cys^11^–Cys^40^ was formed with air oxidation and finally Cys^23^–Cys^41^ disulfide bond was prepared with iodine solution.

### 4.6. Evaluation of Antibacterial Properties

Bactericidal properties of the obtained peptides (HBD-3, direct HBD-3 and [Sec^18,33^]HBD-3) were evaluated against Escherichia coli PCM 2057 and Staphylococcus aureus PCM 2054 strains (purchased from Polish Collection of Microorganisms, PCM) according to the method described by André J. Ouellette and co-workers [52] with slight modifications. Briefly, bacteria cultures grown to exponential phase in Luria Bertani broth (LB) were collected by centrifugation, washed twice and resuspended to a concentration of 108 CFU/mL using 0.5 McFarland standard in the sterile 10 mM PIPES (piperazine-1,4-bis(2-ethanesulfonic acid) buffer, pH 7.4, supplemented with 1% (*v*/*v*) LB (PIPES-LB). Then, the various concentrations of defensins (100 µL) prepared by two-fold serial dilution in PIPES-LB buffer on 96-well microtiter plates were mixed with 100 µL of the adjusted bacterial suspensions containing about 5 × 106 CFU/mL. These samples were incubated stationary at 37 °C for 4 h. After this time, 10 µL of bacterial suspensions pretreated with the tested peptides as well as the control probes: positive (no peptide) and negative (no bacteria) controls from each well were plated in triplicate on the LB agar. Additionally, the aliquots of 10 µL from each well were transferred to the respective wells of fresh 96-well plates containing 190 µL of sterile liquid LB broth. Thus, bactericidal effect of the peptides was confirmed twice: on solid and liquid medium. All plates were incubated for 18–20 h at 37 °C. Minimal bactericidal concentration (MBC) was defined as the concentration of the peptide expressed in µg/mL, which caused complete eradication of bacteria in the initially inoculated medium. All tests were performed in triplicate. We observed similar results of bactericidal effect both for bacteria grown on solid and liquid LB media after pre-treatment with the tested peptides. 

Furthermore, stability in 10 mM PIPES buffer of the tested peptides was performed. Peptides were dissolved in 10 mM PIPES (0.5 mg/mL) and mixed at RT for 24 h. To evaluate their stability in biological buffer, the samples were collected after 0 min, 1 h, 6 h and 24 h, quenched with formic acid and monitored by analytical RP-HPLC.

## 5. Conclusions

This work undertakes the challenging problem of synthesis and oxidative folding of disulfide-rich HBD-3. Based on diverse factors optimized in the synthesis, the use of ChemMatrix resin and pseudoproline dipeptide building blocks largely improved the final yield of the linear peptide. Because of sequential formation of disulfide bonds with Trt/Acm/Mob strategy of Cys residue protection, we were able to fold HBD-3 with the native-like connectivity of the Cys residues. Finally, an isosteric replacement of one disulfide bond with diselenide bond simplified and improved the oxidative folding of HBD-3 while retaining its antimicrobial activity. Our report described the strategy to obtain higher amounts of the properly folded peptide with higher yields, thus enabling further structure-function and pharmacological studies. 

## Figures and Tables

**Table 1:** The yield of chemical synthesis and oxidative folding of HBD-3 and [Sec^18,33^]HBD-3 under different conditions.

Yield [%]	Wang Resin	TentaGel S PHB Resin	HMPB-ChemMatrix Resin	ChemMatrix Resin and Pseudoproline Blocks	[Sec^18,33^]HBD-3	** Mmt/Trt/Acm
purified linear peptide *	10.1%	22.2%	25.5%	38.4%	10.6%	5.4%
native peptide	0.82%	0.73%	1.3%	3.6%	13.5%	0.44%

* purified with formed Se-Se and one S-S bond for [Sec^18,33^]HBD-3 and HBD-3 (Mmt), respectively. ** Mmt/Trt/Acm Cys protected group used with previously optimized synthesis: HMPB-ChemMatrix resin and pseudoproline dipeptide building blocks.

**Table 2:** Antimicrobial activity of the synthesized peptides against *E. coli* and *S. aureus*.

	MBC [µg/mL]	
	*Escherichia coli* PCM 2057	*Staphylococcus aureus* PCM 2054
HBD-3 #1	12.5	12.5
HBD-3 #2	25	25
[Sec^18,33^]HBD-3	25	12.5

## Data Availability

Not applicable.

## Supplementary Materials

The supporting information can be downloaded at: https://www.mdpi.com/article/10.3390/ijms232012562/s1.
