# Supplementary material for: Improving Fmoc Solid Phase Synthesis of Human Beta Defensin 3
Source: Int J Mol Sci. 2022 Oct 19;23(20):12562. doi: 10.3390/ijms232012562 (PMC9603898; doi:10.3390/ijms232012562)
Supplement: Supplementary file 1 [file ijms-23-12562-s001.zip › ijms-1924252-supplementary.pdf]

Supplementary materials

Aleksandra Walewska [1]*, Paulina Kosikowska-Adamus [1], Marta Tomczykowska [1], Bartosz Jaroszewski [1], Adam Prahl [1] and Grzegorz Bulaj [2]

1 Department of Organic Chemistry, Faculty of Chemistry, University of Gdansk, Gdansk, Poland
2 Department of Medicinal Chemistry, College of Pharmacy, University of Utah, Salt Lake City, USA
* Correspondence: aleksandra.walewska@ug.edu.pl

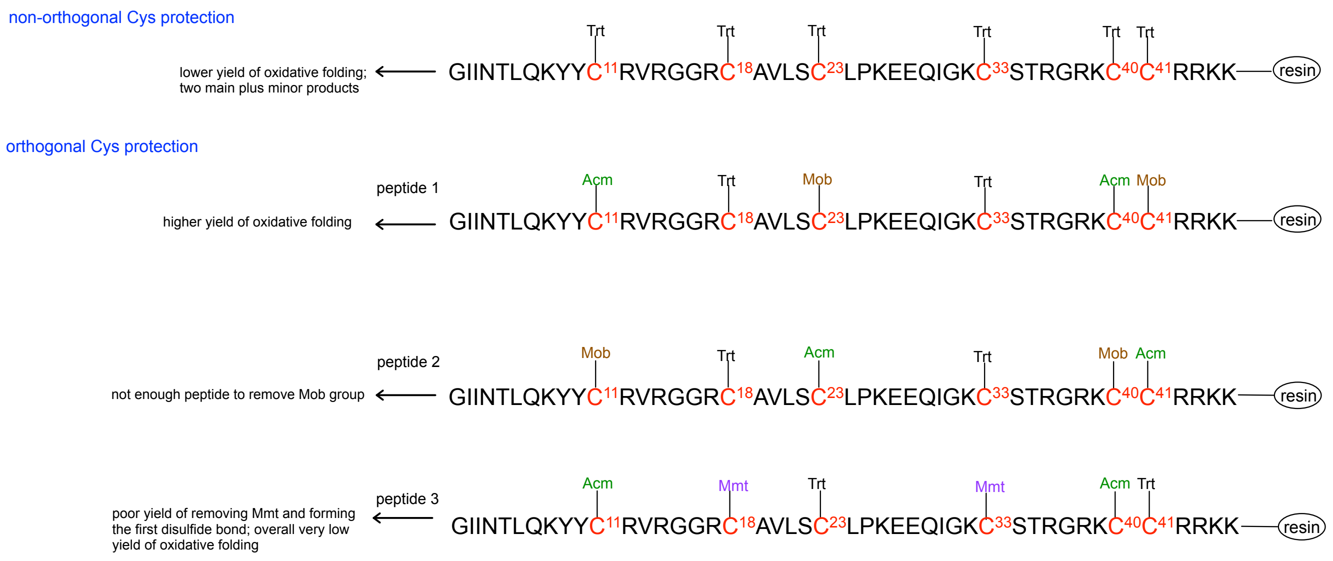

Figure S1. The scheme of pilot syntheses of HBD-3

In silico analysis of HBD-3 sequence

AGGRESCAN is based on an algorithm able to identify a series of protein fragments involved in aggregation. In the plot is shown the predicted relative contribution of individual amino acids to aggregation as defined in AGGRESCAN (arbitrarily normalized to 0). On the X-axis the sequence of amino acids is shown and, on the Y-axis, their respective AGGRESCAN scores. The score above 0 are considered as aggregation prone (shown in red in the plots). The residual score below 0 are meant to be less difficult residues to couple (shown in blue in the plots). The incorporation of pseudoproline dipeptides between Asn^{4}-Thr^{5}, Leu^{21}-Ser^{22} and Ser^{34}-Thr^{35} is shown in green.

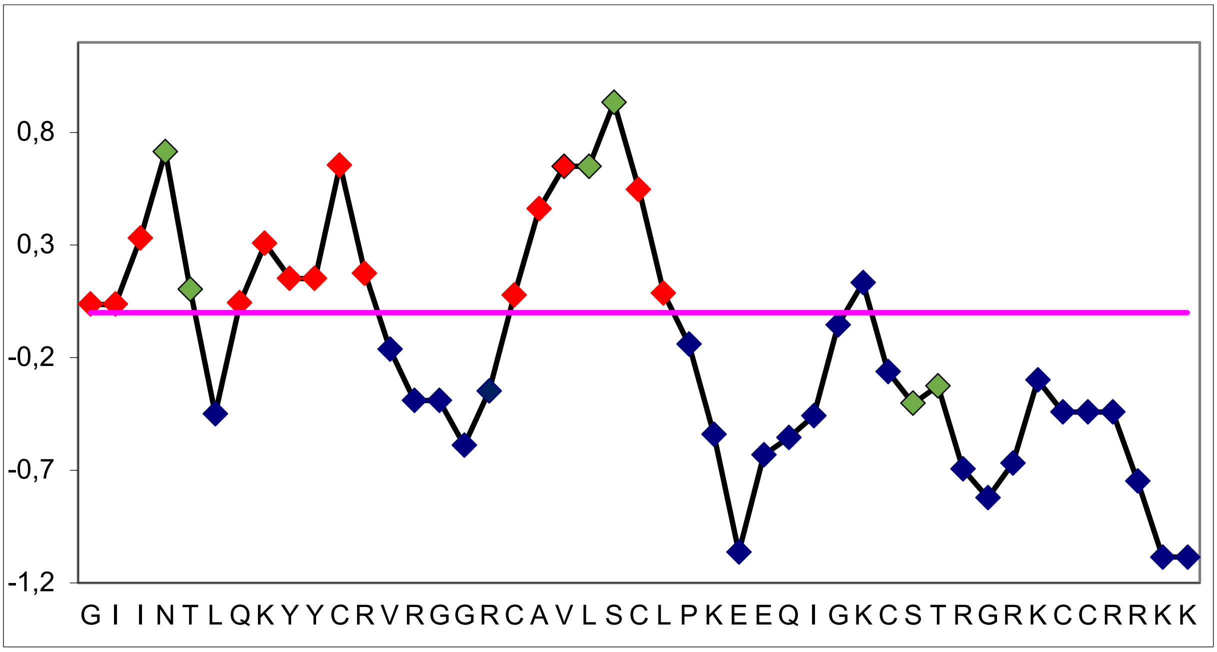

Figure S2. Prediction of aggregation and difficult couplings sections by AGGRESCAN for HBD-3.

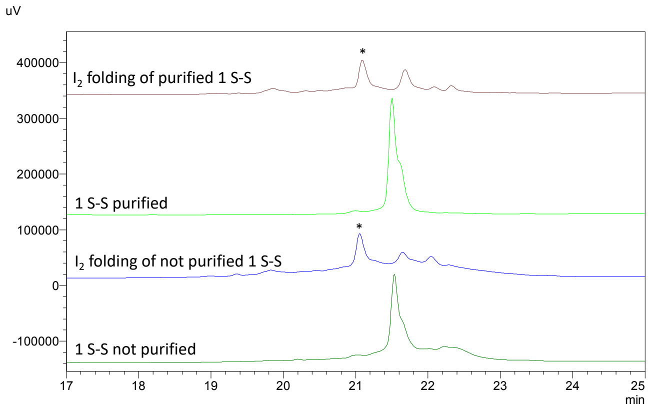

Figure S3. RP-HPLC profiles of oxidative folding with iodine of purified and not purified HBD-3.

MALDI-TOF analysis:

(A) HBD-3 #1, calculated [M+H]$^{+}$: 5156.2 Da, found [M+H]$^{+}$: 5155.7 Da

(B) HBD-3 #2, calculated: [M+H]$^{+}$: 5156.2 Da, found [M+H]$^{+}$: 5155.9 Da

(C) [Sec18,33]HBD-3, calculated: [M+H]$^{+}$: 5249.8 Da, found [M+H]$^{+}$: 5249.7 Da

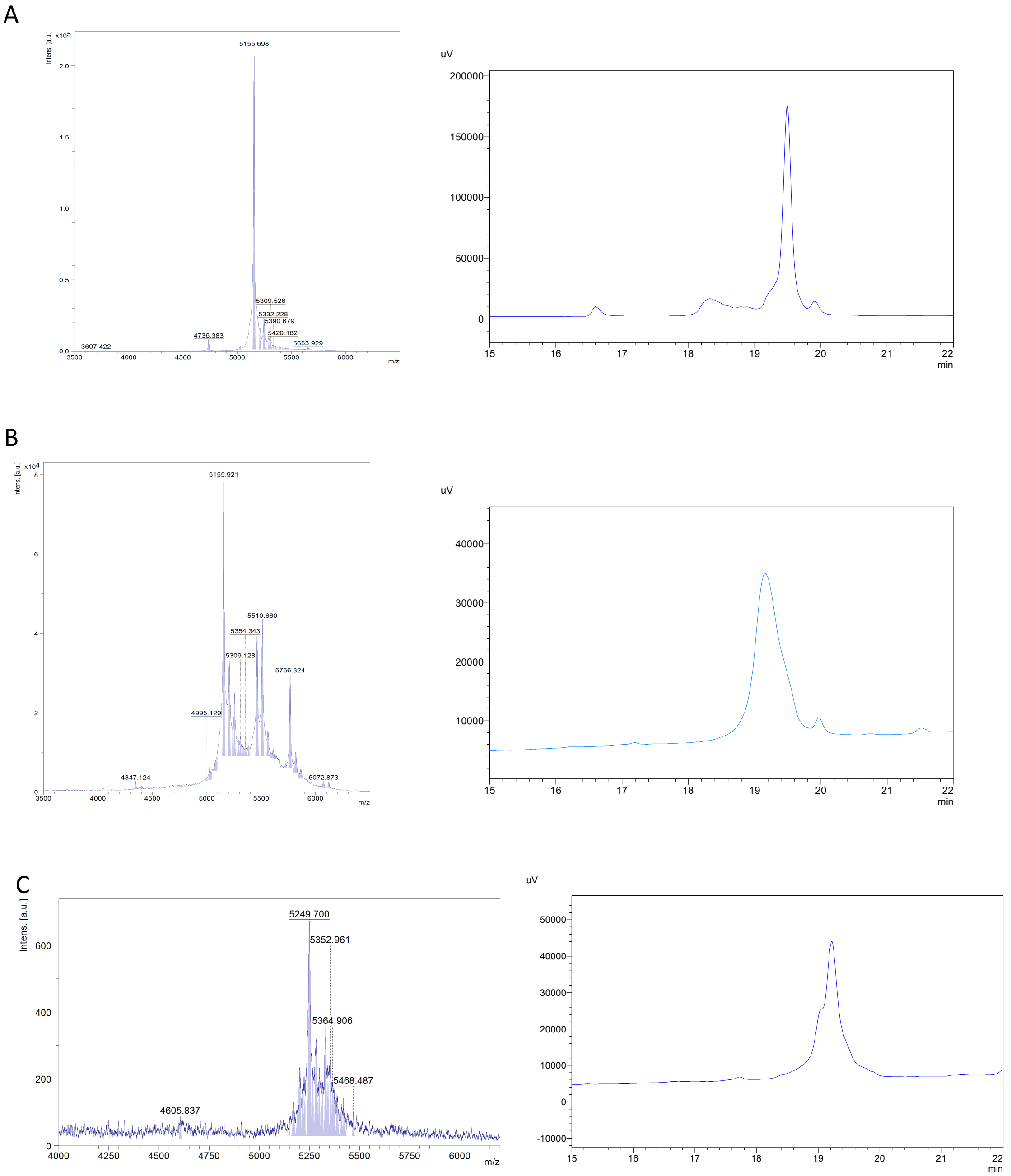

Figure S4. MALDI-TOF spectra and RP-HPLC profiles of synthesized and oxidized peptides. (A) HBD-3 #1; (B) HBD-3 #2; (C) [Sec^{18,33}]HBD-3.

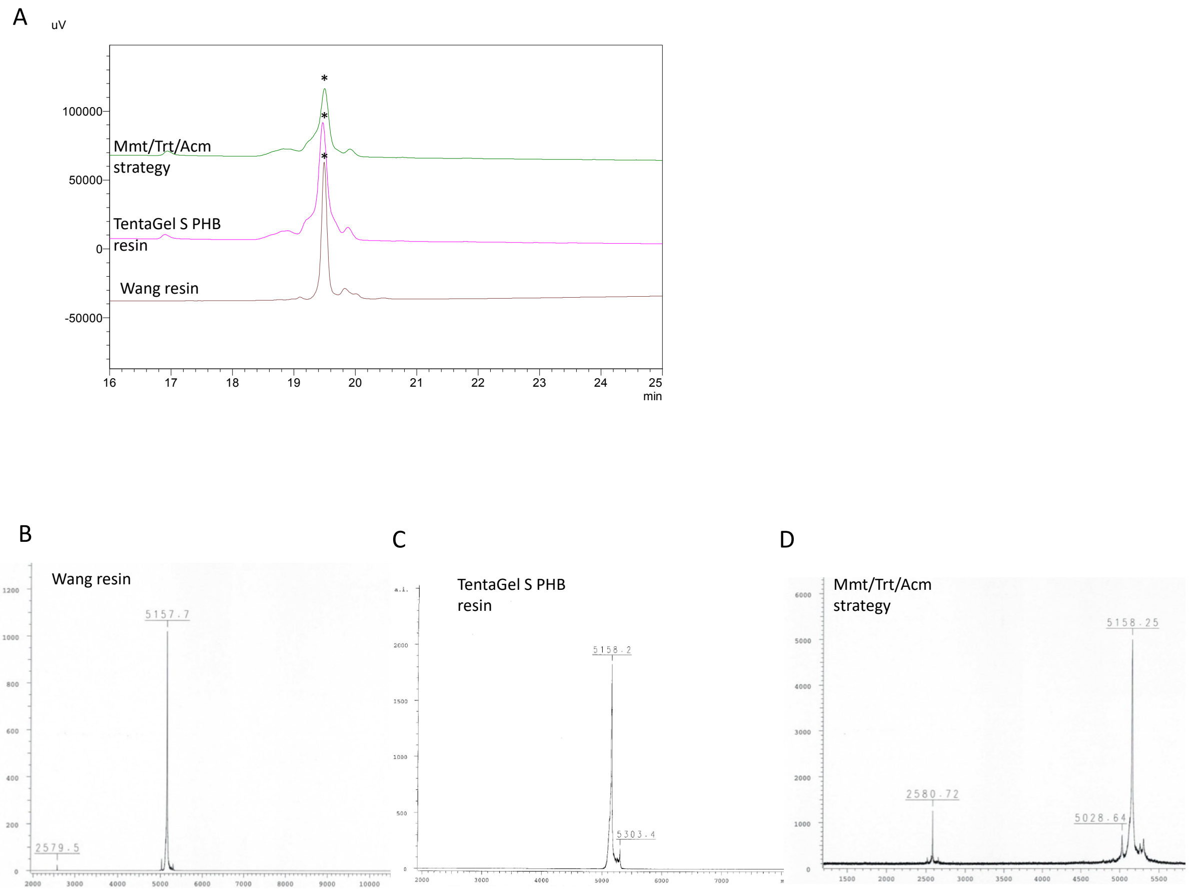

Figure S5. RP-HPLC data (A) and MALDI-TOF spectra of oxidized peptides. (B) HBD-3 synthesized on Wang resin; (C) HBD-3 synthesized on TentaGel S PHB resin; (D) HBD-3 synthesized with Mmt/Trt/Acm strategy

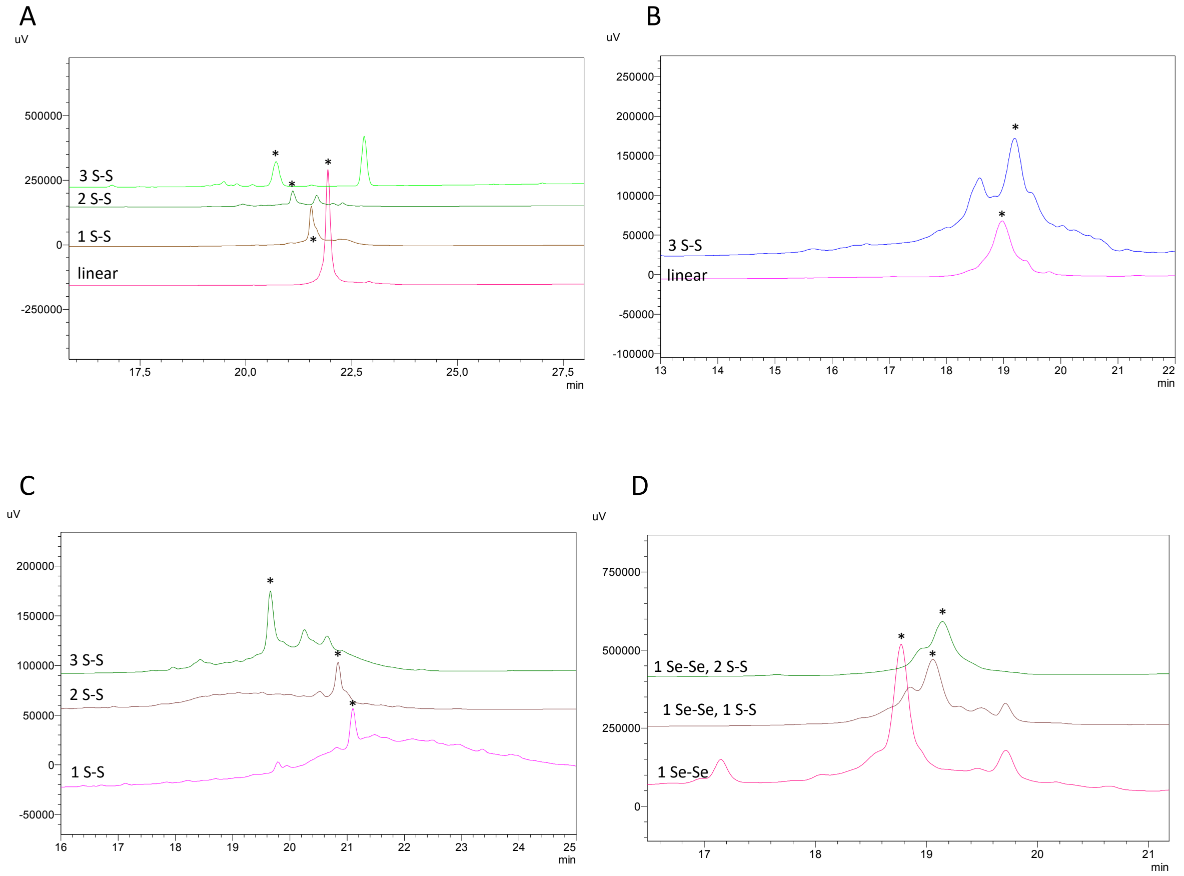

Figure S6. RP-HPLC profiles of disulfide-forming steps of HBD-3: (A) Trt/Acm/Mob strategy; (B) direct folding (C) Mmt/Trt/Acm strategy; (D) diselenide bond strategy

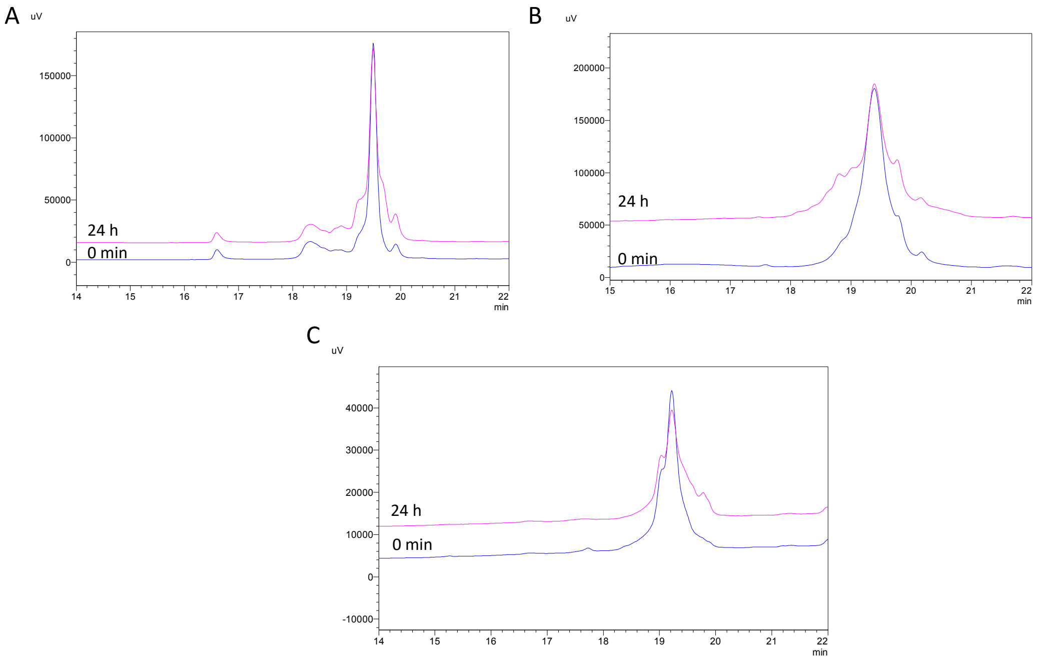

Figure S7: RP-HPLC profiles of synthesized and oxidized peptides carried out in PIPES buffer after 0 min and 24 h. (A) HBD-3 #1; (B) HBD-3 #2; (C) [Sec^{18,33}]HBD-3.

Table S1. Purity of final oxidized products.

**Method of synthesis**	Wang resin	TentaGel S PHB resin	Direct oxidation	ChemMatrix resin and pseudoproline blocks	$[Sec^{18,33}]$HBD-3	Mmt/Trt/Acm strategy
**Purity [%]**	81.2%	79.4%	92.1%	77.2%	83.8%	74.6%
